# Characterization of Cell Glycocalyx with Mass Spectrometry Methods

**DOI:** 10.3390/cells8080882

**Published:** 2019-08-13

**Authors:** Qiongyu Li, Yixuan Xie, Maurice Wong, Carlito B. Lebrilla

**Affiliations:** 1 Department of Chemistry, University of California, Davis, CA 95616, USA; 2 Department of Biochemistry, University of California, Davis, CA 95616, USA

**Keywords:** glycocalyx, glycomics, glycoproteomics, LC-MS/MS, cell membrane proteins, glycosphingolipids, C13 labeling

## Abstract

The cell membrane plays an important role in protecting the cell from its extracellular environment. As such, extensive work has been devoted to studying its structure and function. Crucial intercellular processes, such as signal transduction and immune protection, are mediated by cell surface glycosylation, which is comprised of large biomolecules, including glycoproteins and glycosphingolipids. Because perturbations in glycosylation could result in dysfunction of cells and are related to diseases, the analysis of surface glycosylation is critical for understanding pathogenic mechanisms and can further lead to biomarker discovery. Different mass spectrometry-based techniques have been developed for glycan analysis, ranging from highly specific, targeted approaches to more comprehensive profiling studies. In this review, we summarized the work conducted for extensive analysis of cell membrane glycosylation, particularly those employing liquid chromatography with mass spectrometry (LC-MS) in combination with various sample preparation techniques.

## 1. Introduction

It has long been known that carbohydrates play important roles in disease progression and health maintenance. Unlike proteins, which have DNA as their templates for translation, the synthesis of glycans is a non-template driven process containing competing enzymatic steps. Seven monosaccharides, including mannose (Man), galactose (Gal), glucose (Glc), fucose (Fuc), *N*-acetylglucosamine (GlcNAc), *N*-acetylgalactosamine (GalNAc), and *N*-acetylneuraminic acid (NeuAc), are the building blocks of human *N*- and *O*-glycans on proteins and glycolipids. Glycan synthesis with these monosaccharides in human cells takes place in the endoplasmic reticulum (ER) first and then the Golgi compartment. There are several synthetic pathways in which monosaccharide precursors are provided for glycan synthesis, including salvage and interconversion of monosaccharides. Each type of monosaccharide has a specific nucleotide sugar precursor, such as uridine diphosphate glucose (UDP-Glc) for glucose and guanosine diphosphate mannose (GDP-Man) for a mannose. Nucleotide sugar transporters (NST) are also required for these precursors to be used by glycosyltransferases in both ER and Golgi compartments [1]. Several human disorders are caused by the deficiency of unique transporters [2,3,4,5]. Given that a series of enzymes, including glycosidases and glycosyltransferases, are involved in glycan expression, the inactivation of any of these enzymes by either inhibitors or by gene silencing will also lead to the dysfunction of cells, resulting in the misregulation and folding of proteins, abnormal signal transduction, and even metastasis [6,7,8]. As such, it is important to understand the glycosylation pathways and their products. 

Human cell surfaces contain heavily glycosylated proteins and glycolipids, forming a layer called the glycocalyx. Not only do cell surface glycans act as a protective layer against the exterior environment, but they are also involved in several common cellular processes, such as apoptosis, cell adhesion and migration, and trafficking of membrane glycoproteins. Several studies have been conducted and showed the essential roles that glycosylation play in the protection of cells. A study by Hayashi and Yamashita showed that the glycosylation of the intestinal solute carrier family 26 anion exchanger SLC26A3 was important for cell surface expression and protected the protein from proteolytic digestion. SLC26A3 is a Cl^−^/HCO_3_^−^ exchanger involved in the absorption of Cl^−^, and its mutation leads to diarrhea [9]. Another study demonstrated a similar function of glycosylation where high mannose glycans protected lysosomal membrane proteins from degradation [10]. Furthermore, the tolerance of intestine epithelial host cells (IEC) to mild pathogens was improved by rapid fucosylation, which might be used for the protection of IEC [11]. The glycosylation is also involved in cell apoptosis. For instance, the glycosylation of death receptors, such as cluster of differentiation 95 (CD95), plays key roles during cell apoptosis in its binding to CD95 ligands [12]. Although the function of sialylation on CD95 has not been fully elucidated, several studies have shown that varying sialylation can either inhibit [13] or enhance [14] CD95-induced apoptosis. Furthermore, glycan alterations on cell surface glycoproteins can vary cell adhesion and migration properties. It was demonstrated that the increase of integrin β1 subunit sialylation increased the adhesion of HL60 cells [15]. Besides, the inhibition of fucosylation with 2-fluorofucose suppressed the migration of HepG2 liver cancer cells [16]. Bacterial infection has also been found to alter the glycosylation of mucins on the epithelial cell surface, affecting the expression of both sialic acid and fucose on this heavily glycosylated extracellular protein [17]. In addition to the cellular functions described above, glycosylation also guides the proper folding of membrane proteins further affecting their functions and trafficking [18]. It was demonstrated that the mutation on one glycosylation site of the membrane protein dipeptidyl peptidase IV at Asn319 drastically diminished the activity of the protein. The protein was further retained in cytoplasm and degraded faster after the mutation [19]. The role of glycosylation in trafficking has been studied for several other proteins, such as the bile salt export pump ATP-binding cassette, sub-family B member 11 (ABCB11), H, K-ATPase β subunit, and glycine transporters [20,21,22].

Given that cell surface glycosylation is involved in essential biological functions, and the decrease or increase of specific glycoforms are involved in diseases, they can be regarded as a source of disease biomarkers. Aberrant glycosylation is a hallmark of cancer [23]. Increased expression of truncated *O*-glycans, including Tn and sialyl-Tn antigens, were discovered previously in different tumor tissues [24,25]. Elevated sialylation level was found in individuals with oral potentially malignant disorders (OPMDs) [26]. Noda et al. [27] revealed that the up-regulation of GDP-l-fucose could be used as a potential biomarker in hepatocellular carcinoma (HCC). The function of core-fucosylation was investigated by Wang et al. [28]. They showed that the deficiency of *N*-glycan core-fucosylation on transforming growth factor β1 (TGF-β1) receptor in mice resulted in destructive emphysema. Another study on core-α-1,6-fucosylated triantennary (NA3Fb) glycans illustrated that the level of NA3Fb was increased on HCC tumor cell surface and also in HCC patient serum samples [29]. Enhanced expression levels of several glycoproteins, including versican, galectin-3, and periostin, was found in ovarian tumor tissues compared to normal tissues [30]. In aggressive prostate tumor tissues, the co-regulation of cathepsin L and a variety of extracellular membrane (ECM) proteins, such as periostin and MFAP4 (Microfibrillar-associated protein-4), was observed [31]. The underglycosylated mucin 1 (MUC-1) antigen is known to be overexpressed in different human epithelial cell adenocarcinoma, such as ovarian and pancreatic cancers, and it can be targeted for cancer therapy [32,33,34]. Other studies have shown that the shedding of gangliosides is one typical characteristic of human medulloblastoma and neuroblastoma tissues [35,36]. The monosialic ganglioside (GM3) is found with elevated levels in lung cancer tissues [37]. Furthermore, a glycosphingolipid (GSL) expression is altered during the progression of colorectal cancer [38]. 

Although the combination of monosaccharides gives a large number of possible glycan structures [39], only a limited number of structures are found naturally due to limitations in glycosyltransferases. In humans, there are hundreds of unique glycan compositions. Also, linkages between each monosaccharide in a glycan vary not only in terms of linkage position but also in α or β anomer-types, which makes the extensive analysis of glycosylated compounds difficult. Nonetheless, large strides have been made through various strategies.

The simplest technique for obtaining structural information is to use lectins or monosaccharide binding antibodies [39,40,41]. Lectins have different binding specificities, for example, concanavalin A (ConA) binds to high-mannose type *N*-glycans, while *Sambucus nigra* agglutinin binds to *N*-glycans with α-2,6 linked sialic acids. They can be used to image cells and in enzyme-linked immunosorbent assays (ELISA) [42]. However, lectins are not applicable for comprehensive glycan analysis. With the development of cell membrane extraction methods and mass spectrometric techniques, more detailed and sensitive analyses of cell surface glycosylation have been achieved. Glycans released from glycoconjugates with enzymatic or chemical methods are separated by liquid chromatography (LC). Analysis with mass spectrometry provides accurate masses, while fragmentation methods can be used to determine glycan compositions. Furthermore, site-specific analysis using intact glycopeptides is now achievable with the development of new software and high-performance mass spectrometry. 

**Figure 1:**
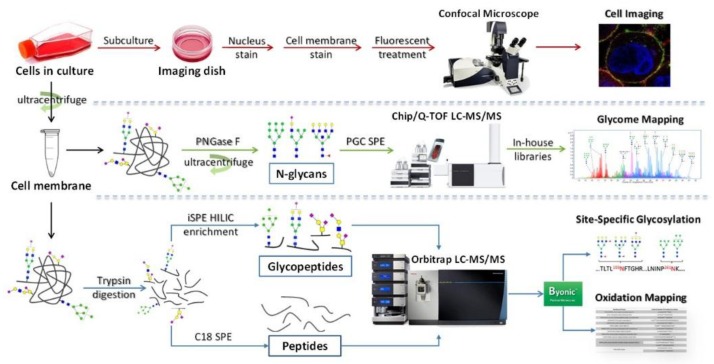
A workflow for the comprehensive analysis of cell membrane glycans and proteins with LC-MS/MS and confocal imaging. (Abbreviations: PNGase F: Peptide-*N*-glycosidase F; PGC: porous graphitic carbon; SPE: solid phase extraction; HILIC: hydrophilic interaction liquid chromatography.)

In this review, we summarized the advances made recently in the analysis of cell surface glycosylation using liquid chromatography coupled to mass spectrometry (LC-MS). We also described related preparatory techniques, such as cell membrane extraction and glycopeptide enrichment. The applications of these techniques in elucidating the roles of glycosylation in cellular functions and disease progression were also reviewed (Figure 1). 

## 2. Cell Membrane Extraction Methods

The comprehensive characterization of the cell surface glycocalyx requires specific and efficient methods for extracting cell membrane from the cell lysates. There are currently numerous strategies to enrich the cell membrane that are mainly divided into two categories based on analyzing either a specific target component or a broader set of fractions containing several components [43].

When the enrichment of a specific target is desired, affinity enrichment through non-covalent interactions between specific protein-ligand pairs can be employed [44]. Lectin-based affinity and antibody-mediated immunoaffinity are two of the most common methods used [45,46]. Lectins are carbohydrate-binding proteins that have preferences for certain glycan moieties, while antibodies belong to a class of proteins that can bind to specific glycan antigens. Thus, the membrane fraction containing specific targets of lectins or antibodies can be selectively immobilized or precipitated, and the specific recognition event leads to the enrichment of the desired components. Given that the glycome and proteome vary among different types of samples, it is important to select the proper lectin or antibody for enriching the desired cell plasma membrane proteins. Another strategy is to provide specific handles for bioorthogonal target enrichment through metabolic labeling of cell surface glycans with unnatural glycans [47]. Research groups of Bertozzi [48] and Wu [49] have enriched and identified cell surface proteins containing sialylated and fucosylated glycans by introducing into cells modified monosaccharide analogs that act as enrichment tags. It is anticipated that the availability of newer probes would enable more specific enrichment of plasma membrane proteins from complex biological matrices.

Given the amphiphilicity of the cell plasma membrane, biphasic separation through polyethylene glycol (PEG) and Triton X-114-based methods are also used to extract cell membrane [50,51]. Among them, the PEG-dextran system is the most extensively used. Because the PEG phase is more hydrophobic and less dense compared to the dextran phase, the cell plasma membrane can be enriched at the upper PEG layer [52]. Although this method provides efficient separation of the cell membrane, the difficulties of detergent removal have generally limited subsequent mass spectrometry analysis. Detergents complicate the separation of *N*-glycan, *O*-glycans, and glycolipids from other cellular components and can interfere with analyte detection. Kim et al. demonstrated a method in which cationic colloidal silica beads were introduced to the cells in culture [53]. The negatively charged plasma membrane can firstly be captured through their electrostatic interactions with the beads, and further isolated using ultracentrifugation. However, this method has thus far only be applied for cultured cells [54]. 

Enrichment tag methods, such as biotinylation of the cell membrane, have also been reported [55]. Biotin tags are conjugated to the cell surface, followed by enrichment with streptavidin and recovery of the plasma membrane. Care must be taken to evaluate the proper biotin tag candidates. To selectively label the cell plasma membrane, membrane-permeable biotin reagents, such as succinimidyl 2-(biotinamido)-ethyl-1,3′-dithiopropionate (NHS-S-S-biotin), should not be used. Pan et al. have successfully utilized and modified the membrane-nonpermeable biotin reagent, sulfosuccinimidyl-20 (biotinamido)ethyl-1,3-dithiopropionate (Sulfo-NHS-S-S-biotin), to label cell membrane glycoproteins for further enrichment and analysis [56]. Cowell et al. introduced a traceless biotin tag, which forms a (2-(alkylsulfonyl)ethyl) carbamate upon bioconjugation and allows sulfonyl-triggered release [57]. However, the reaction efficiency varies among different proteins, and biotinylated lysine may lead to missed-cleavage in the downstream tryptic digestion [58]. This drawback can be minimized using other reagents, such as carbonyl-reactive biotin. Carbohydrates on glycoproteins can be oxidized by periodate treatment, and the biotin tag can be conjugated using hydrazide-based chemistry [59]. However, this method results in the loss of information regarding the detailed glycan structures and prevents the comprehensive characterization of cell surface glycoproteins. Wang et al. developed a non-covalent labeling method using cholesterol–PEG–biotin, in which the cell plasma membrane was biotinylated through cholesterol insertion via hydrophobic interaction [60]. 

A more common method for membrane enrichment uses a series of differential centrifugation to extract the membrane from cell and tissue lysates [61]. Suski et al. reported a technique using a discontinuous sucrose gradient to fractionate plasma membrane from the crude membranes containing plasma membrane, plasma membrane-associated membranes, and endoplasmic reticulum membrane [62]. Consequently, different fractions were separated in several bands depending on the contrastive densities of the membranes. The plasma membrane could be found at the interface with the sucrose gradient ranging from 43% to 53%. Lund et al. took advantage of the low viscosity and low osmolarity of Percoll to create a Percoll/sucrose density separation technique, which allowed for the more efficient identification and quantification of metastasis-associated cell surface markers [63]. The results revealed that this method had less contamination from other fractions because the plasma membrane could be directly obtained from the top layer. Similar to this approach, the discontinuous Nycodenz/sucrose solution was developed to extract the plasma membrane from cells [54]. 

**Figure 2:**
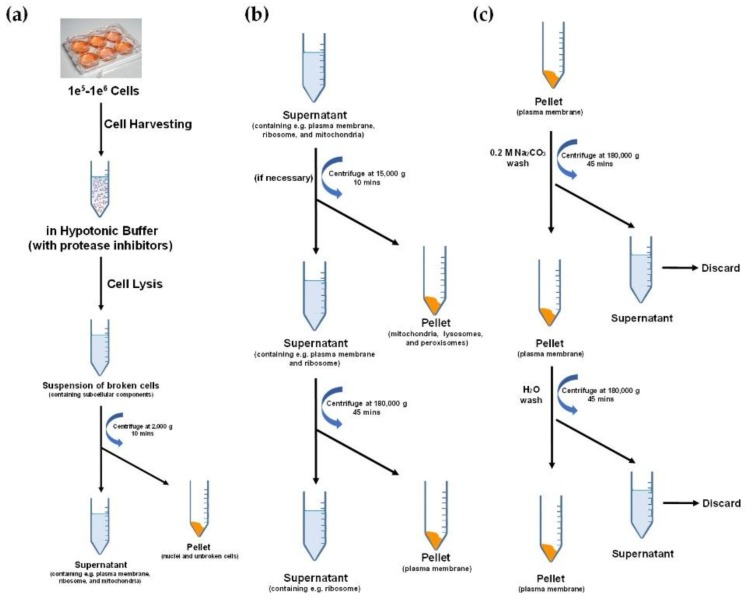
The fractionation of plasma membrane with hypotonic buffer through following steps: (**a**) Cell lysis and separation of nuclei and unbroken cells; (**b**) Plasma membrane extraction through ultracentrifuge; (**c**) Plasma membrane wash with Na_2_CO_3_ and H_2_O.

However, the procedural complexity and the high background contamination associated with these methods can affect the subsequent characterization of the glycocalyx. Successive fractionation steps give high specificity but may lead to loss of material. This loss is exacerbated when the amount of sample is limited. To circumvent these issues, we developed a simpler cell membrane protein enrichment method with compatibility and applicability for mass spectrometry-based glycomic and glycoproteomic analyses (Figure 2) [64]. In this approach, the cell or tissue samples were mixed with hypotonic buffer containing sucrose and protease inhibitors. The nucleus could be removed by centrifugation at low speed, and the cell membrane fraction could be pelletized using ultracentrifugation. Sodium carbonate was then used to purify the enriched membrane. In this way, the loose plasma membrane-associated membranes could be dissolved by the high alkalinity of sodium carbonate solution. At the same time, cell plasma membrane proteins, such as integral membrane proteins, remained insoluble in the carbonate solution [65]. The pellet was washed thereafter one more time with deionized water to remove the remaining sodium carbonate, and the resulting membrane pellet was ready for further analysis. 

## 3. Glycomic Analysis of Cell Membrane

### 3.1. Methods for Cell Membrane Glycan Analysis

Both enzymatic and chemical methods have been used to release glycans from glycoproteins and glycolipids. For example, peptide-N-glycopeptidase F (PNGase F) is the most commonly used enzymes for releasing mammalian *N*-glycans because of its broad specificity and good activity among different types of *N*-glycans. For *N*-glycans with α-1,3-core fucosylation or core xylose, such as those found in invertebrates and plants, respectively, another enzyme, PNGase A, is employed. However, sialylation can affect the effectiveness of PNGase A. Several other endoglycosidases with different substrate specificities are commercially available. Endoglycoceramidases (EGCase) I and II can be used to cleave glycans from GSLs. EGCase II can effectively cleave ganglio-, lacto-, and neolacto- type GSLs, but display low to zero activity towards certain types of GSLs, such as globo- type GSLs and fucosyl GM1 [66]. While EGCase I enzyme has a broader specificity, which cleaves both ganglio- and globo- type GSLs. Chemical methods can also be used to release glycans from GSLs. Ozonolysis-based chemical release of glycans under relatively mild condition has been applied to GSLs. Glycan analysis has shown higher yields compared to other chemical methods [67]. Currently, there is no known enzyme with broad substrate specificity that can cleave *O*-glycans from peptides or proteins. Chemical methods, such as reductive alkaline β-elimination, are used for glycan release after which alditols are generated. However, the method can come with undesirable by-products, and the harsh conditions can lead to loss of certain moieties, such as *O*-acetylation. 

It is necessary to separate glycans from intact proteins or lipids for further analysis. Lectins of varying specificities have been widely used to capture glycosylated proteins for enrichment. Following glycan release, the proteins are readily washed away, and the glycans are analyzed directly [68,69]. With these techniques, only certain types of glycans can be analyzed due to the limited specificity of lectins. Another approach for glycan purification is with solid-phase extraction (SPE). Given that glycans contain a large number of hydroxyl groups, they can be separated from other more hydrophobic molecules using hydrophilic interaction liquid chromatography (HILIC) [70]. It has been found that the derivatization of glycans yields better enrichment [71]. Glycans can also be retained with porous graphitic carbon (PGC). PGC cartridges are now commonly used to desalt and clean up glycans before MS analysis [72]. 

Different derivatization methods can be applied to increase the ionization efficiency of glycans for MS analysis or make them suitable for other detectors, such as fluorescence [73]. One of the commonly used labeling methods employs reducing end derivatization and reductive amination with compounds, such as 2-aminobenzamide (2-AB) and 2-aminobenzoic acid (2-AA). The labeled glycans are quantifiable with a UV detector [74]. They can also be quantified with mass spectrometry [75,76]. Derivatization with 1-phenyl-3-methyl-5-pyrazolone (PMP) through Michael addition reaction introduces two amine groups, thereby enhancing the ionization efficiency in MS analysis [77,78]. The analysis of native glycans without derivatization are also commonly performed [79,80,81]. Although the chromatogram is more complicated due to separated peaks of α and β anomers of underivatized glycans, loss of glycans caused by further manipulation is avoided so that more comprehensive results are obtained.

After sample preparation and glycan isolation, purified glycans can be analyzed with various combinations of chromatography, ionization methods, and mass spectrometry instrumentation. One method is to use matrix-assisted laser desorption/ionization (MALDI) coupled with an accurate mass detector, such as time-of-flight (TOF), or Fourier-transform ion cyclotron resonance (FTICR). The MALDI source tolerates higher salt and contaminant levels, and the use of high-resolution FTICR MS enables the identification of more glycan compositions. Using a MALDI-FTICR, De Leoz et al. [82] performed the *N*-glycomic analysis of native glycans in a prostate cancer cell line pRNS and the sera of prostate cancer patients. With the derivatization of native glycans through permethylation or esterification, the in-source fragmentations from MALDI can be eliminated, making it more compatible with high-resolution MS, including FTICR [77]. MALDI-MS has been extensively applied to analyzing derivatized glycans released from biological samples. Hung et al. [83] employed MALDI-TOF to the characterization of permethylated or benzimidazole-derivatized polysaccharides. In another study, MALDI-TOF-MS was utilized to analyze sialylated *N*-glycans after esterification [84]. With the derivatization, linkages of sialic acids have also been characterized. 

However, the application of MALDI source is limited by its incompatibility with chromatographic separation, which allows comprehensive glycan profiling with isomer separation. The employment of electrospray ionization (ESI) provides the ability to analyze more complicated samples by coupling it to liquid chromatograph (LC). Through ionizing analytes with multiple charges, a wider mass range is obtained. Additionally, nano-flow LC coupled to nano-ESI source provides higher sensitivity, allowing characterization of low abundance glycans. Thus, nanoflow liquid chromatography, electrospray ionization, and time-of-flight MS (nanoLC-ESI-TOF-MS), as an analytical platform, has been extensively used for biomarker discovery, as well as performing functional studies. More applications are elaborated with details in the following subsection. 

Previous part discusses mainly the *N*-glycan analysis. Here we have also talked briefly about the challenges in *O*-glycan analysis. In contrast to *N*-glycans, the difficulty of structural analysis of *O*-glycans firstly lies in the lack of consensus sequence of *O*-glycosylation sites. Besides, *O*-glycans have eight types of core structures [85] with varied extensions, making their analysis more complicated than the analysis of *N*-glycans, which have three types of core structures. Furthermore, currently, there is no universal enzyme to cleave *O*-glycans, especially those with extended and complicated structures. One enzyme for *O*-glycan release but with critical specificity is endo-GalNAc-ase. It can only be used for the cleavage of disaccharides with Core 1 (Gal β1,3GalNAc) structure [86]. The existing chemical methods for cleaving *O*-glycans include reductive β-elimination [87], non-reductive release [88], and oxidative release [89], from where not only released *O*-glycans are obtained, some by-products are also generated. Some types of *O*-glycans, such as *O*-GlcNAc, the abundance of which is highest in the nucleus, are difficult to be detected mostly because it is sub-stoichiometric at each modification site [90]. More details about the analysis of *O*-GlcNAc have been reviewed by Ma et al. [91]. Despite the above issues, advances have been made in this area. The comprehensive characterization of *O*-glycans for diseases and biomarker discovery has been achieved by analyzing chemically released *O*-glycans with MALDI-FTICR [92]. The nanoLC-TOF-MS/MS has also been employed to profile *O*-glycans on human glycoproteins that are involved in the interaction with “siglec-like” binding regions on cell surfaces of *Streptococcus gordonii*, a type of human oral microbiota [93]. Several other studies learning the structures of *O*-glycans and their functions in cancer cellular processes have been conducted before [94,95]. 

### 3.2. Application of Glycomic Analysis with LC-MS/MS

An and coworkers [96] analyzed the cell surface glycosylation of human embryonic stem cells (hESC) with nanoLC-MS analysis by enriching cell membrane with ultracentrifugation. The *N*-Glycan analysis revealed that the dominant type on hESC was high mannose glycans, which was also validated with lectin assays and flow cytometry. Changes in *N*-glycosylation during the maturation process of intestinal cell line Caco-2 was studied by Park et al. [97]. Caco-2 cell line has long been used as an established in vitro model for studying absorptive intestinal epithelial cells [98]. The quantitative results demonstrated that levels of fucosylated and sialylated glycans were increased, while the level of high-mannose type glycans was decreased during differentiation. The results were consistent with PCR analysis, showing that the genes encoding mannosidases were up-regulated together with the gene *MGAT3*, which is responsible for producing bisecting GlcNAc glycans, and the gene *B4GALT3*, which encodes for a galactosyltransferase. 

**Figure 3:**
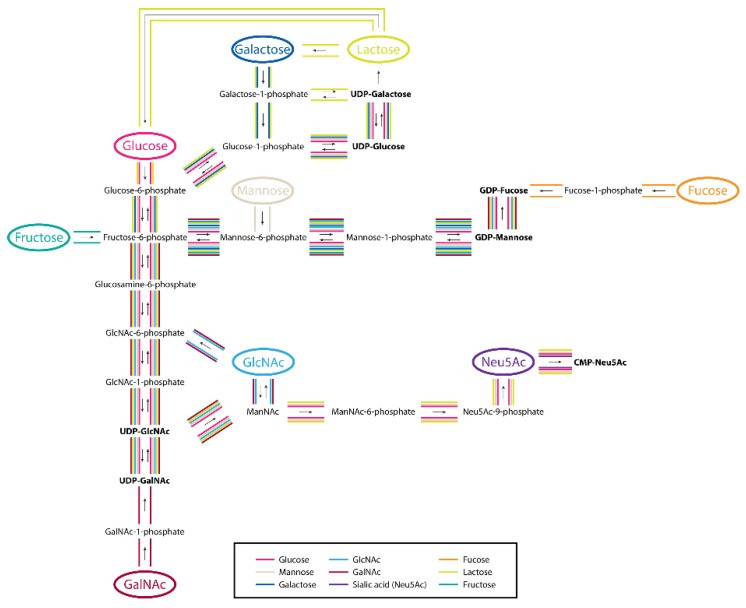
The metabolic pathways of different human dietary monosaccharides and their interconversions. Reprinted with permission from ref. 78. Copyright 2017 Oxford University Press.

The external environment of the intestine luminal space can vary for multiple reasons. It is important to know how environmental changes affect cell surface glycosylation to understand the influence of glycan variation on cellular function. One study has been conducted on in vitro intestine models, including cell lines Caco-2 and HT-29, to learn the effects of alterations in the cell culture conditions on glycan expression (Figure 3) [99]. Here, the cultured cells were separately provided with nine different exogenous dietary monosaccharides, several types of short-chain fatty acids, and grown in various pH values, followed by the characterization of released cell membrane *N*-glycans. It was found that supplementation with different exogenous monosaccharides had varied effects on the *N*-glycan expression of both cell lines. Treatment with short-chain fatty acids (SCFAs), including acetate, lactate, and butyrate, which are byproducts of gut microbe, resulted in significant changes in fucosylated *N*-glycans for both cell lines. Besides, the culture condition with lower pH altered the *N*-glycan types with a striking increase in sialylated glycans. The sensitivity of intestinal epithelial cell lines to different conditions of the external environment was elucidated through the *N*-glycosylation profile, indicating that the culture condition should be considered when using cell lines. 

Cell surface glycans are interaction sites in the defense against foreign bodies. It has long been recognized that host cell surface glycans are involved in microbe infection and invasion. The glycosylation changes of host cells after infection with *Salmonella typhimurium* with different coincubation times were studied previously using cell line Caco-2 [100]. During the time course study, the redistribution of glycosylation was found after 1-h infection, where high-mannose glycans increased significantly. The use of kifunensine, the mannosidase inhibitor, which leads to the increased expression of high mannose glycans, was consistent with the notion that the adherence and invasion of bacteria were enhanced by high-mannose glycans. Besides, the abundances of sialylated species decreased after infection. The linkage study using different exoglycosidases demonstrated further that species containing the α-2,3-linked sialic acid decreased in abundances. Both observations were due to the presence of sialidases expressed by the bacteria. 

The function of core-fucosylation produced by fucosyltransferase 8 (FUT8) was investigated by Awan et al. [101]. They showed that the migration of multipotent stromal cells (MSCs) was promoted by the protein fibroblast growth factor (FGF2) through the triggering of FUT8 expression. The cell membrane glycomic analysis illustrated that the level of core fucosylation on cell surface *N*-glycans was increased. On the other hand, the silencing of FUT8 in two biological models both resulted in the restriction of *N*-glycan movement in protein integrin, which further reduced the migration of cells. 

## 4. Glycoproteomic Analysis of Cell Membrane 

The glycoproteomic analysis provides simultaneous analysis of both glycans and proteins. Despite recent developments in mass spectrometry techniques, the analysis of intact glycopeptides is still challenging. One of the issues is the diminished abundances of individual glycopeptides owing to the microheterogeneity at each glycosite. Compared to peptides, glycopeptide analysis requires further enrichment due to ion suppression from the more ionizable peptides. Glycopeptides can be enriched with techniques, such as lectin affinity chromatography [102] and boronic acid-functionalized silica [103]. Metabolically labeled glycopeptides containing functional groups, such as azido groups [104,105] and alkyne groups [106,107], can be enriched with cross-linker modified biotin and streptavidin. However, these approaches are all applicable to only specific types of glycopeptides, and the introduction of unnatural monosaccharides may perturb the cell status in unexpected ways. For a more generalized and comprehensive study, hydrazide beads have been employed to enrich glycopeptides nonselectively [108]. The limitation of this technique is that the glycans must be cleaved from peptides. Furthermore, the analysis is limited by the reduced efficiency of PNGaseF release due to steric hindrance [109]. The analysis of intact glycopeptides can be enhanced with HILIC enrichment. The performance of three different types of HILIC solid phases for enriching glycopeptides derived from human plasma was assessed previously, and electrostatic repulsion hydrophilic interaction liquid chromatography using strong anion exchange-electrostatic repulsion-hydrophilic interaction chromatography (SAX-ERLIC) solid-phase extraction provided the most extensive coverage of N-linked glycopeptides [110].

Glycosylated proteins can also be separated by SDS-gels with subsequent glycoproteomic analysis of isolated fractions. In one example, the glycosylation study was conducted on the serum samples collected from patients with ovarian cancer and ovarian cancer cell lines [111]. Rather than analyzing the changes in the whole *N*-glycan compositions, the glycosylation on the gel-separated individual glycoproteins, including immunoglobulin A1, apolipoprotein B-100, and fibronectin, were profiled and compared. 

Another challenge in the confident identification of intact glycopeptides is the difficulty in fragmenting both the peptide backbone and the glycan appendage effectively with common tandem-MS methods. Peptide bonds and glycosidic bonds fragment through different mechanisms and at different energies. Given that low energy collision-induced dissociation (CID) methods fragment mainly the glycan moiety of a glycopeptide while preserving the peptide backbone relatively intact, other alternatives are needed. Compared to low energy CID, high-energy collisional dissociation (HCD) methods yield more fragmentations on the peptide backbones [112]. With stepped HCD collision energy, intact glycopeptides can be characterized with better coverage of both glycans and peptides after the enrichment [113]. 

In contrast to HCD and CID, electron transfer dissociation (ETD) fragments peptide backbones more readily than the glycans of glycopeptides [114]. By combining ETD and HCD, where ETD fragments glycopeptides mainly along the peptide backbone to yield c ions and z ions and HCD along with the glycan structure, more comprehensive fragmentation spectra can be obtained [115]. However, the lengthened cycle time due to the shuttling of the precursor ion for electro-transfer/higher-energy collision dissociation (EThcD) fragmentation may result in the loss of glycopeptide identification due to the lower number of glycopeptides probed [116]. The employment of triggered fragmentation making use of the oxonium ions from glycan fragments to trigger the process may make the enrichment unnecessary for glycoproteomic analysis [117]. 

The improvements in the glycoproteomic analysis also rely on the continuing development of software. Currently, the most commonly used commercial software for analysis is Byonic (Protein Metrics), where the digestion of proteins with different enzymes can be selected, and various types of modifications can be searched [118]. Peptides are simultaneously searched against a proteome database, and glycan fragments are matched to a glycan composition library to give both the glycopeptide and glycoprotein identities. Diverse fragmentation methods, including CID, HCD, ETD, and EThcD, can be incorporated into the search engine. Another applicable software developed by Liu et al. is called pGlyco, which can search for glycopeptides fragmented with stepped HCD [119]. The software has been applied to glycoprotein standard mixtures as well as cell and tissue samples. In these experiments, the glycopeptides were enriched with ZIC-HILIC cartridges [120]. The software also has the capability of searching triggered MS^3^ spectra of glycopeptide precursors. Other software have been developed for glycoproteomic analysis and include Integrated Glycoproteome Analyzer (I-GPA) [121], SweetNET [122], and gFinder [123].

By applying these techniques of intact glycopeptide analysis, Park et al. [64] investigated the cell surface sialylation by labeling sialylated glycans metabolically with *N*-azidoacetyl-mannosamine (ManNAz) while determining where the unnatural azido *N*-acetylneuraminic acids (SiaNAz) were incorporated. *N*-glycopeptides were enriched with two types of HILIC cartridges, including zwitterionic (ZIC)-HILIC and iSPE-HILIC. With stepped collision energy HCD, more than 2000 and 500 nonredundant glycoforms were identified for Caco-2 and PNT2 cell lines, respectively. They determined that glycoproteins with sialylation tended to have more occupied *N*-glycosites. The incorporation of SiaNAz further showed site-specificity.

**Figure 4:**
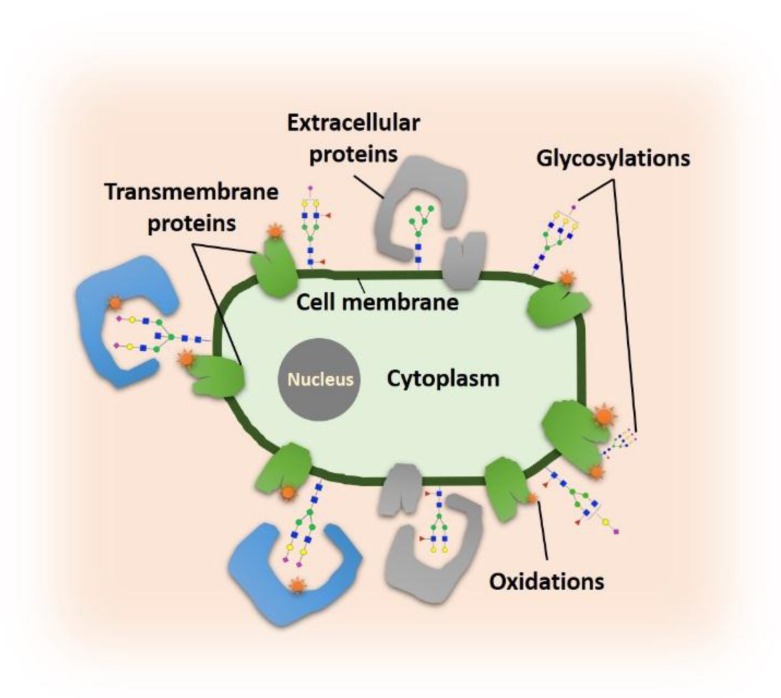
The POSE (protein oxidation in the sialic acid environment) tool is used for characterizing sialic acid-interacting proteins on the cell membrane or extracellular membrane. Reprinted with permission from ref. 103. Creative Commons license (CC BY 3.0) (http://creativecommons.org/licenses/by/3.0/).

The interactions of glycoproteins with *cis*- or *trans*- targets are drawing more attention given that cell surface glycoproteins play essential roles in a variety of biological functions. These interactions often involve sialic acid-binding proteins, such as siglecs, and different types of lectins, highlighting the importance of cell surface sialylation. The identification of these binding proteins is challenging and is performed almost always empirical. To enhance the identification of sialic acid-binding proteins, Li et al. [124] developed a proximity labeling method aimed at the mapping of protein oxidation in the sialic acid environment (POSE) through the functionalization of cell surface sialylated proteins and the covalent labeling of sialic acid-binding proteins with radicals (Figure 4). Following the metabolic incorporation of SiaNAz by treating cells with ManNAz, the synthesized Fe(III) probe, DBCO-FeBABE (Dibenzocyclooctyne-functionalized p-Bromoacetamidobenzyl-EDTA, iron (III) chelate), was chemically conjugated to the SiaNAzylated glycoproteins. The hydroxyl radicals were generated from H_2_O_2_ with Fe(III) acting as the catalyst. The reaction led to the self-oxidation of sialylated glycoproteins and the oxidation of sialic acid-binding proteins, such as LAMA5 (laminin subunit α-5) and L1CAM (L1 cell adhesion molecule). The POSE method provides a novel tool for the discovery of proteins that interact with sialic acid, which can be further applied to understand cell-cell and cell-microbe interactions. 

## 5. Glycosphingolipids Analysis of Cell Membrane

Glycosphingolipids (GSLs) are composed of a glycan headgroup covalently linked to a sphingolipid tail. Due to the variety of structures of both parts, the number of possible GSL structures according to different biosynthesis pathways can number in the tens of thousands [125,126]. For complete structural elucidation of GSLs, traditional separation strategies, including thin-layer chromatography or liquid chromatography, can be used [127,128]. With affinity assays, such as lectin or antibody, GSL with the relevant glycosylation type can be identified and quantitated [39,129,130]. However, affinity-based methods are limited to GSLs, which express certain glycan epitopes, and no information about the lipid can be obtained. 

Progress has been made in the comprehensive identification of GSLs with better sensitivity due to recent developments in mass spectrometry. With tandem MS, the compositions and sequences of both glycans and lipids can be elucidated. However, linkage elucidation in glycan headgroups still relies on enzymatic digestion with exoglycosidases before MS analysis. The employment of endoglycosylceramidases (EGC) reduces the degree of complexity from ceramides so that the glycomic analysis can be conducted on headgroups [131,132] However, the separate analyses of glycans and ceramides do not provide correlated information on both parts.

To address this issue, the comprehensive analysis of intact GSLs with techniques, such as LC-ESI-MS and MALDI MS, has been developed and applied to different biological samples. Gangliosides in human serum have been characterized before with nanoLC-MS, and the alterations related to pancreatic cancer have been observed [133]. As a common nutrition source for humans, milk contains a significant amount of GSLs, which can have important health implications [134]. The GSL contents and types of ovine milk and bovine milk have been studied, and the binding properties of certain types of GSLs to different microbes have been revealed [135,136]. Studies have also revealed that cell membranes are highly enriched in GSLs, especially in microdomains, such as lipid rafts [137]. Various properties and functions of cells, including cell-cell adhesion and signal transduction, are mediated by GSLs. GSLs account for a high abundance among lipids in the nervous system, and they play essential roles in brain development [138]. Previously, mouse brain gangliosides were studied by Zarei et al. [139] with MALDI-TOF, and the method was optimized for sialylated gangliosides. Ion mobility MS has recently been used to add another dimension of separation of isomeric gangliosides isolated from the human hippocampus [140,141]. In another study, GSLs from animal brains with Gaucher disease (GD) were quantitatively analyzed with LC-MS/MS for understanding the neuropathology [142]. 

**Figure 5:**
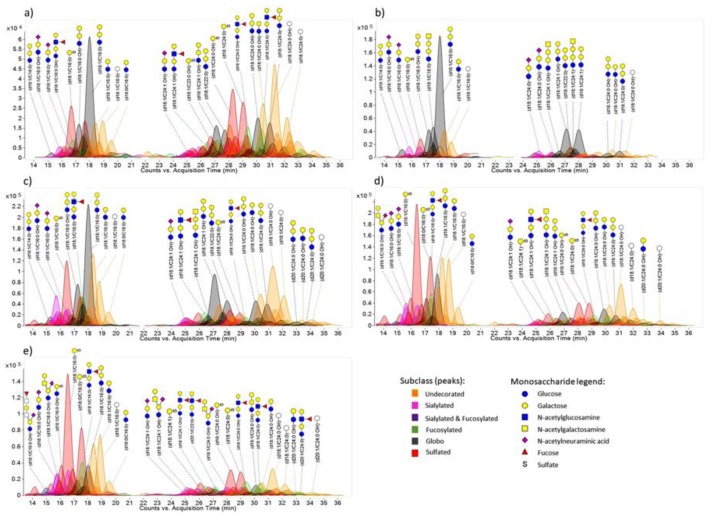
GSLs (glycosphingolipids) chromatograms of Caco-2 cell line at different stages of confluency and differentiation (**a**) Day 5, (**b**) Day 7, (**c**) Day 14, (**d**) Day 21, and (**e**) Day 24. Reprinted with permission from ref. 122. Creative Commons license (CC BY 4.0) (http://creativecommons.org/licenses/by/4.0/).

Recently, Wong et al. [143] developed a method for extensive identification and quantitation of cell membrane GSLs with nano HPLC-chip-Q-TOF MS, with which more than 200 intact GSLs of differentiated Caco-2 cell line were characterized (Figure 5). With tandem MS, the composition and connectivity of glycan head groups were profiled, together with their attached ceramides with varying numbers of hydroxyl groups, lipid lengths, and degrees of unsaturation. The application of the method to the Caco-2 cell line demonstrated that GSLs with sialylated and sulfated head groups increased, while globo-type GSLs decreased, during differentiation. Furthermore, the ceramides with 32 to 34 carbons showed elevated relative abundances during the maturation, while those with longer lengths ranging from 40 to 42 carbons decreased. The method can be applied to the GSL analysis of tissue samples.

## 6. Glycosylation Studies with Isotopic Labeling

### 6.1. Metabolic Pathways of Glycans

The measurement of glycolytic pathways, gluconeogenesis, and glycogenolysis using isotope labeling and their contributions to metabolic biology have been extensively reviewed in the past several years [144,145,146]. However, except for extensive studies of glucose, very little is known about the relative or absolute contributions to de novo and salvage pathways of other monosaccharides. Here, we offered a brief overview of recent developments of techniques in the study of biosynthesis and interconversion of monosaccharides and their products using stable isotope-labeled precursors.

It has been recently discovered that mannose supplementation has a significant role in therapeutics by slowing cancer growth in mice, and it also enables the suppression of autoimmune diabetes [147,148]. Previously, Ichikawa et al. utilized ^13^C isotope-labeled glucose and mannose to determine the origins of mannose in glycoproteins [149]. At physiological levels of glucose and mannose (5 mM and 50 uM, respectively), mannose was efficiently utilized, and up to 45% of mannose was directly provided in *N*-glycans. Interestingly, when the concentration of mannose increased to 1 mM, mannose became the general source of all the products, including lactate, pyruvate, alanine, galactose, and *N*-acetyl-glucosamine, in *N*-glycans, although it was still not utilized for glycogen synthesis. It was found that phosphomannose isomerase activity and exogenous mannose concentration determined the metabolic flux of mannose into the *N*-glycosylation pathway.

The metabolic pathways for synthesizing GDP-Fucose have already been elucidated [150]. Early studies in HeLa cells suggest that the de novo pathway, in which GDP-mannose is converted to GDP-fucose by a series of enzymes, is the major source of GDP-fucose, while a minor salvage pathway contributes less than 10% [151]. However, the early work was limited in scope and was unable to account for fucose at physiological concentrations. A more comprehensive study by Ng et al. demonstrated that the salvage pathway of fucose was preferred for fucosylated *N*-glycans when fucose was available at physiological levels [152]. Furthermore, the salvage pathway suppressed the de novo pathway, especially for the production of GDP-Fucose, because it did not alter the contributions of other monosaccharides, including galactose and mannose, in *N*-glycans. This strongly supports that the metabolic pathway of GDP-fucose is very specific. 

Recently, Xu et al. used ^13^C uniformly labeled monosaccharides to investigate the incorporation pathways of different dietary sugars in *N*-glycans and glycopeptides [153]. The variance of monosaccharide utilization and their interconversion among different cell lines were determined, and the rate of monosaccharide incorporation was found to be glycan-specific and protein-dependent. The methods described here can be effectively applied to unravel the contributions of different metabolic pathways, which may provide us an opportunity to rethink old concepts and identify new mechanisms. 

### 6.2. Glycan Quantitation Using Isotope Labeling 

Although quantitative glycomics may be performed without labeling, the comparative relative quantitation of glycans can be enhanced with isotope labeling methods [154]. With this strategy, two or more glycan samples, each labeled with a unique number of heavy isotopes, can be analyzed in the same run, thus avoiding batch variations. A simple and straightforward strategy is based on the permethylation of glycans using isotopically labeled reagents [155]. Reductive amination has also been used for the reducing end of glycans with conjugating isotope-labeled compounds, such as aniline [156], 2-aminobenzoic acid (2-AA) [157], 4-phenethylbenzohydrazide [158], and Girard’s reagent [159]. More recent approaches improve the quantitation of specific glycans. For example, ^13^C labeled p-toluidine can be used to quantify sialylated glycans making use of the carboxylic acid group contained uniquely in sialic acid [160]. Although the sialylated glycans can be specifically labeled using 1-ethyl-3-(3-dimethylaminopropyl) carbodiimide (EDC)-catalyzed coupling reactions, the method is only applicable for purified glycan pools. Recently, Wei et al. demonstrated the duplex stable isotope labeling (DuSIL) method to distinguishably quantify sialylated and neutral *N*-glycans [161]. 

**Figure 6:**
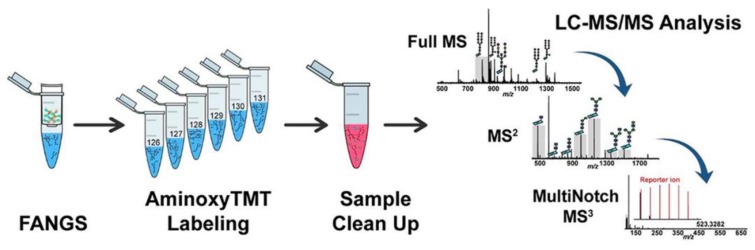
The workflow of MS^3^ analysis of multiplex isobaric tag labeled *N*-glycans. Reprinted with permission from ref. 147. Copyright 2018 American Chemical Society.

The capacity of analysis with isotope labeling is shown to be impeded by the high spectral complexity and the requirement of ultra-high resolving power MS [162]. The introduction of novel isobaric tags enables multiplexed glycan analysis, such as isobaric aldehyde reactive tags (iARTs) [163], quaternary amine-containing isobaric tag for glycan (QUANTITY) [164], aminoxy tandem mass tag (aminoxyTMT) [165], glycan reductive isotope-coded amino acid labeling (GRIAL) [166], and glycan reducing end dual isotopic labeling (GREDIL) [167]. Occasional issues arise, however, such as relatively low reporter ion yield for labeled complex glycans. Chen et al. [168] have applied additional MS^3^ scans using the nanoHILIC-Tribrid quadrupole-ion trap-Orbitrap system to characterize samples prepared in what they termed as filter-aided-*N*-glycan separation (FANGS). The method yielded improved accuracy, precision, and sensitivity (Figure 6). An isobaric multiplex reagent for carbonyl-containing compound (SUGAR) with high ion yield has also been introduced by Li and co-workers recently [169]. Barrientos and Zhang have recently demonstrated the isobaric labeling of intact gangliosides, but the method is not applicable for nonsialylated GSLs [170]. While these labeling strategies are widely used for the relative quantitation of labeled glycans among samples, no absolute quantitation is achievable. These methods also come with the caveat that the accuracy of relative quantitation is highly dependent on the incorporation of the label, namely these methods strictly require high modification efficiency and specificity of the labels.

To address this problem, enzymatic methods have been applied to modify stable isotope-labeled glycans. Zhang et al. introduced PNGase F-catalyzed glycan ^18^O-labeling (PCGOL) for relative glycan quantitation, in which PNGase F was utilized to incorporate ^18^O into *N*-glycans [171]. Shi et al. have successfully used mutant enzyme Endo-M-N175Q to label glycans with the stable isotopic label by transglycosylation reaction [172]. Reichardt and co-workers constructed a ^13^C-labeled complex *N*-glycan library containing 15 synthesized glycan isotopologues with a mass shift of 8 Da through chemo-enzymatic methods for absolute glycan quantification [173]. By quantifying every glycan with its corresponding heavy isotope-labeled internal standard, the glycans of a monoclonal therapeutic antibody were quantified with excellent speed and accuracy. With the recent developments in chemoenzymatic glycan synthesis, it is anticipated that the isotope-labeled glycan library can be enlarged with better glycan coverage for glycan quantitation [174,175,176].

### 6.3. Glycopeptide Quantitation Using Isotope-Labeling 

Recent advances in the development of various glycoproteomic analysis strategies allow for more confident identification and quantification of glycopeptides based on mass-to-charge ratios of the intact glycopeptide and fragment ions generated by different types of activation methods [177]. Several quantification methods based on stable isotope labeling have been used in combination with glycoproteomic approaches, including chemical labeling, stable isotope dimethyl labeling, and metabolic labeling [178].

Chemical labeling techniques provide efficient quantitation for glycoproteomic analysis. Examples include isobaric tags for relative and absolute quantitation (iTRAQ) and tandem mass tags (TMT), which use a family of isobaric isotope compounds to label the N-terminus of peptides generated from tryptic protein digests [179]. Zhang et al. first employed the iTRAQ method for glycoproteomic analysis combined with ^18^O stable isotope labeling, which allows for the identification of *N*-glycosylation sites and glycopeptide quantitation [180]. In their method, four sample groups were analyzed simultaneously, where the *N*-glycosites of two sample sets were labeled with H_2_^16^O, and two other samples were labeled with H_2_^18^O through PNGase F cleavage. Meanwhile, all peptides from the four biological samples were labeled with four iTRAQ reagents in parallel. By this approach, labeled peptides were identified by LC-MS/MS, and both glycopeptides and deglycosylated peptides could be quantified at the same time. Additionally, the glycosylation site occupancies were also determined. Zhang and co-workers successfully quantified over 1800 unique *N*-glycopeptides corresponding to over 600 *N*-glycoproteins from prostate cancer cell lines using iTRAQ labeling [181]. Kalxdorf et al. used TMT labeling to monitor dynamic changes of the cell surface glycoproteins and to gain mechanistic insights into macrophage differentiation [182]. The multiplexing capacity of such assays is expected to be improved further to be applied to biological samples with high complexity. 

An alternative method is stable-isotope dimethyl labeling by reductive amination, which was first introduced to quantitative proteomic analysis with high reaction yield and reproducibility [183]. Combined with various glycopeptide enrichment strategies, stable-isotope dimethyl labeling has been further extended for glycopeptide quantitation [184]. Weng et al. developed an integrated platform using HILIC enrichment and dimethyl labeling for quantitative *N*-glycoproteomic analysis [185]. Lin et al. introduced microcrystalline cellulose to separate glycosylated and non-glycosylated peptides simultaneously, and used formaldehyde-H2 and -D2 to label the two fractions [186]. The resulting glycopeptides were quantified by ESI-ion trap based on peptide concentration and glycan profile. Zou and co-workers developed a labeling method to enrich the glycopeptides through hydrazide beads, and the glycopeptides were quantified through stable isotope labeling [187]. They showed high detection sensitivity of the method during quantitative glycoproteomics analysis, where 42% of the annotated glycosites were quantifiable using only 10 μg of standard glycoprotein mixtures [188]. They further improved their method with peptide N-terminal protection (PNP) strategy to minimize the sample loss from undesired covalent bonding to the beads. Consequently, the glycoproteomics coverage was largely increased [189].

**Figure 7:**
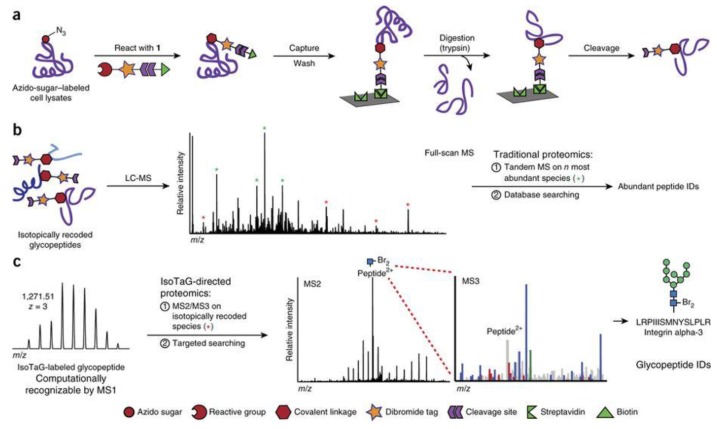
The workflow of IsoTaG (Isotope Targeted Glycoproteomics) enrichment method: (**a**) the enrichment of labeled glycoproteins; (**b**) the LC-MS/MS analysis of isotopically relabeled glycopeptides; (**c**) data analysis using targeted searching. Reprinted with permission from ref. 169. Copyright 2015 Springer Nature.

Woo and co-workers developed a metabolic labeling method, called Isotope Targeted Glycoproteomics (IsoTaG), to characterize and quantify intact glycopeptides by MS (Figure 7) [190]. The cell culture samples were first labeled with a bioorthogonal probe, followed by the enrichment with an isotopic recoding affinity probe. Intact glycopeptides were recovered by cleavage of the probe, and the IsoTaG-labeled glycopeptides could be computationally recognized by MS1 scan and triggered for further tandem mass spectrometry (MS^2^) and MS^3^ fragmentations. The combination of efficient biotin enrichment strategy and targeted mass-independent data analysis enabled extensive quantitation of samples. IsoTaG was successfully employed to map and quantify azido-bearing sialylated glycans [191], and the method was extended to analyze alkyne-labeled glycopeptides [192]. Most recently, over 2000 O-linked glycopeptides from human T cells were identified and quantified [193]. However, due to the varied incorporation efficiency of the bioorthogonal probe across glycoproteins, the application of IsoTaG to quantitation is limited to comparing the same glycoprotein from the same cell line. 

Compared to label-free quantitative glycoproteomics, labeling methods possess the following advantages. First, due to the capability of simultaneous analysis of samples, labeling methods, such as iTRAQ and TMT, require less instrument time. These techniques also provide the high accuracy of quantitation [194]. Besides, experimental variations are eliminated when conducting metabolic or chemical labeling quantitation. On the other hand, the applications of some labeling methods are limited to a small number of samples [195], making them not suitable for clinical analysis. The sample preparation procedures of some labeling techniques can be time-consuming [196]. It has also been demonstrated that label-free quantitation provides a higher dynamic range, ranging from three to four orders of magnitudes [196,197,198]. By considering all the aspects mentioned above, the suitable quantitative approaches could be chosen when conducting quantitative glycoproteomic analysis. 

## 7. Conclusions and Future Directions

The cell surface glycocalyx is essential to various cellular functions, and there are correlations between changes in the glycocalyx and many diseases, such as cancer, immune deficiencies, and cardiovascular disease. Hence, characterization of the cell membrane glycosylation, especially with mass spectrometry-based glycomic and glycoproteomic analyses, is of wide and considerable interest. Extensive advancements in mass spectrometry and sample preparation methods have been made, but further improvements are required to propel the field towards more widespread adoption. Reliable glycan and glycoprotein standards and well-characterized reference materials are still needed to build consensus among different research groups. Furthermore, developments in bioinformatics tools can enable rapid-throughput quantitation of native, labeled, and isotopically labeled glycans and glycoproteins. With continuous breakthroughs in the field, we anticipate that the analysis of cell surface glycocalyx with mass spectrometry would make more significant contributions to disease diagnosis and therapeutics.
